# SARS-CoV-2 Affects Thyroid and Adrenal Glands: An ^18^F-FDG PET/CT Study

**DOI:** 10.3390/biomedicines11112899

**Published:** 2023-10-26

**Authors:** Chiara Lauri, Giuseppe Campagna, Andor W. J. M. Glaudemans, Riemer H. J. A. Slart, Bram van Leer, Janesh Pillay, Marzia Colandrea, Chiara Maria Grana, Antonio Stigliano, Alberto Signore

**Affiliations:** 1 Nuclear Medicine Unit, Department of Medical-Surgical Sciences and of Translational Medicine, Sant’Andrea University Hospital, “Sapienza” University of Rome, 00161 Rome, Italy; giuseppe.campagna@uniroma1.it (G.C.); alberto.signore@uniroma1.it (A.S.); 2 Department of Nuclear Medicine and Molecular Imaging, University Medical Center Groningen, 9713 GZ Groningen, The Netherlands; a.w.j.m.glaudemans@umcg.nl (A.W.J.M.G.); r.h.j.a.slart@umcg.nl (R.H.J.A.S.); b.van.leer@umcg.nl (B.v.L.); 3 Department of Critical Care, University Medical Center Groningen, University of Groningen, 9713 GZ Groningen, The Netherlands; j.pillay@umcg.nl; 4 Nuclear Medicine Division, European Institute of Oncology—IRCCS, 20141 Milan, Italy; marzia.colandrea@ieo.it (M.C.); chiara.grana@ieo.it (C.M.G.); 5 Endocrinology, Department of Clinical and Molecular Medicine, Sant’Andrea University Hospital, “Sapienza” University of Rome, 00161 Rome, Italy; antonio.stigliano@uniroma1.it

**Keywords:** COVID-19 patients, SARS-CoV-2, thyroid gland, adrenal glands, ^18^F-FDG PET/CT, ICU

## Abstract

Background: Since most endocrine glands express ACE-2 receptors and can be infected by SARS-CoV-2 virus, this retrospective multicentre observational study aims to assess the metabolic activity of thyroid and adrenal glands of COVID-19 patients by ^18^F-FDG PET/CT. Methods: We retrospectively evaluated the ^18^F-FDG PET/CT scans of COVID-19 patients admitted by three different centres, either in a low-intensity department or in the intensive care unit (ICU). A visual assessment and a semi-quantitative evaluation of areas of interest in thyroid and adrenal glands were performed by recording SUVmax and SUVmean. The ^18^F-FDG PET/CT uptake in COVID-19 patients was compared with those observed in normal age-matched controls. Results: Between March 2020 and March 2022, 33 patients from three different centres (twenty-eight patients in a low-intensity department and five patients in ICU), were studied by ^18^F-FDG PET/CT during active illness. Seven of them were also studied after clinical remission (3–6 months after disease onset). Thirty-six normal subjects were used as age-matched controls. In the thyroid gland, no statistically significant differences were observed between control subjects and COVID-19 patients at diagnosis. However, at the follow-up PET/CT study, we found a statistically higher SUVmax and SUVmean (*p* = 0.009 and *p* = 0.004, respectively) in the thyroid of COVID-19 patients. In adrenal glands, we observed lower SUVmax and SUVmean in COVID-19 patients at baseline compared to control subjects (*p* < 0.0001) and this finding did not normalize after clinical recovery (*p* = 0.0018 for SUVmax and *p* = 0.002 for SUV mean). Conclusions: In our series, we observed persistent low ^18^F-FDG uptake in adrenal glands of patients at diagnosis of COVID-19 and after recovery, suggesting a chronic hypofunction. By contrast, thyroid uptake was comparable to normal subjects at disease onset, but after recovery, a subgroup of patients showed an increased metabolism, thus possibly suggesting the onset of an inflammatory thyroiditis. Our results should alert clinicians to investigate the pituitary–adrenal axis and thyroid functionality at the time of infection and to monitor them after recovery.

## 1. Introduction

Since the outbreak of coronavirus disease 2019 (COVID-19) pandemic, increasing evidence in the literature highlights that severe acute respiratory syndrome coronavirus 2 (SARS-CoV-2) does not show a selective tropism on the respiratory system. It is, indeed, now well known that SARS-CoV-2 variants have widespread effects on many tissues and organs, including central nervous, cardiovascular and digestive systems, thus shifting COVID-19 from a respiratory syndrome to a systemic disease [1,2]. A large body of literature also describes endocrine gland impairment deriving from several factors: a direct gland damage induced by the virus, an indirect effect on hypothalamus–pituitary gland axis, a massive production of inflammatory cytokines and chemokines and virus-triggered inflammation [3,4].

SARS-CoV-2 mainly uses angiotensin-converting enzyme 2 (ACE-2) receptors to gain cellular access, causing tissue damage [3]. ACE-2 receptors are variably expressed in pituitary, thyroid, adrenal, pancreatic and gonadal glands; therefore, the endocrine system offers several potential targets for viral-induced damage [5,6,7]. It is now clear that endocrine disorders contribute to the complex and varied symptoms experienced by infected patients and that some of them may take a long time to normalize endocrine functionality [5,6,7].

It has been reported that 13 to 64% of COVID-19 patients are affected by thyroid disfunctions [4], with a higher prevalence compared to non-COVID-19 subjects [8,9,10]. Transient thyrotoxicosis has been mainly described in patients admitted to the intensive care unit (ICU) [11] and it has been associated with high interleukin 6 (IL-6) levels [12]. This would increase cardiovascular risks, such as arrhythmias, that have been frequently reported in COVID-19 patients. Sub-acute thyroiditis and Graves’ thyrotoxicosis have also been described [13,14] as well as hypothyroidism, with the majority of patients reverting to normal thyroid function after several months post infection [8].

Similar findings have been described after SARS-CoV-2 vaccination, thus suggesting a direct role of spike protein interaction with ACE-2 receptors, cross reactivity with thyroid proteins and immune-mediated phenomena triggered by the vaccine itself [15,16,17].

Adrenal impairment has also been frequently reported. Its aetiology is multifactorial. Several case reports described adrenal insufficiency due to adrenal infarction or haemorrhage [18,19,20,21,22]. Moreover, it has been postulated that SARS-CoV-2 is able to impair the stress-induced cortisol production by expressing several amino acid sequences that mimic human adrenocorticotropic hormone (ACTH). Therefore, the host’s antibodies produced against the virus will also cross-react and inactivate endogenous circulating ACTH [23,24]. This “immune-invasive strategy” may result in the development of corticosteroid insufficiency and could predispose to more critical clinical presentation of respiratory tract infection and persisting symptoms [23,24]. In addition to this, a direct injury on the adrenal cortex, due to the expression of ACE2 receptors in the *zona fasciculata* and *reticularis*, impairs the glucocorticoid synthesis [25]. The indiscriminate use of high doses of exogenous corticosteroids, which have been largely used for therapy, determines a suppression of hypothalamus–pituitary–adrenal (HPA) axis, thus contributing to hypocortisolism [23,26,27,28,29].

As the acute and devasting phase of SARS-CoV-2 pandemic is passed, “long COVID” is becoming the new challenge facing clinicians. This condition, due to a multi-systemic involvement, is characterized by persistent malaise, fatigue, dizziness, myalgia and joint pain, headache and possible cognitive disturbances affecting the patient for a long time after COVID-19 diagnosis [30,31,32]. It has been postulated that adrenal insufficiency concurs in the development and maintenance of fatigue, myalgia and arthralgia, which have been reported in 65%, 50.6%, and 54.7%, respectively, of long COVID patients [33,34]. Nevertheless, data on cortisol levels in COVID-19 patients are rarely reported and provide discordant findings [34,35] and, in general, the long-term impact of endocrine dysfunction still needs to be further elucidated.

Fluorine-18 fluorodeoxyglucose positron emission tomography/computed tomography (^18^F-FDG PET/CT) is not recommended for diagnosis or for follow-up of COVID-19 patients despite the literature having extensively described incidental cases of SARS-CoV-2 infection diagnosed in patients undergoing ^18^F-FDG PET/CT for other reasons [36,37,38,39,40].

Moreover, with the increasing awareness that SARS-CoV-2 infection is not only confined to the respiratory tract, but rather showing a multi-systemic tropism, several papers have described the ^18^F-FDG uptake in extra-thoracic tissues and organs during SARS-CoV-2 infection or vaccination [41,42,43,44,45,46,47,48,49]. During the early months of the COVID-19 pandemic, after observing the first two patients affected by SARS-CoV-2 infection [42], we performed a qualitative and semi-quantitative analysis of ^18^F-FDG uptake in several organs/tissues, in addition to lungs, and correlated these measurements with patients’ haematological parameters [43]. Given the increasing evidence of endocrine gland involvement, our attention now focuses on the analysis of ^18^F-FDG uptake in the thyroid and adrenal glands. Therefore, the aim of this retrospective multicentre observational study is to describe ^18^F-FDG uptake in thyroid and adrenal glands in COVID-19 patients at baseline, during follow-up, and in comparison to normal age-matched subjects.

## 2. Materials and Methods

### 2.1. Patients

Inclusion criteria for patients in this retrospective study were:

− Positivity to nasopharyngeal swab with real time polymerase chain reaction (RT-PCR) test for SARS-CoV-2 between 2020 and 2022;
− Patients admitted in low intensity departments or intensive care unit (ICU);
− Availability of at least one ^18^F-FDG PET/CT scan during the active SARS-CoV-2 infection performed in order to assess the extent of the disease.

Exclusion criteria were:

− Patients with pre-existing structural and/or functional alterations of thyroid and adrenal glands;
− Patients who previously received chemotherapy or biologic therapies for oncologic reasons;
− Pregnancy or nursing women;
− Age < 18 years.

For the control group, we retrospectively evaluated ^18^F-FDG PET/CT studies performed for several oncologic and non-oncologic clinical indications between 2020 and 2022. Patients without a history of thyroid and adrenal dysfunctions, based on the electronic patient file, and showing a normal ^18^F-FDG biodistribution without any pathological uptake were enrolled. Patients receiving chemotherapy or immune check-point inhibitors at the time of the study were excluded from the analysis.

### 2.2. The ^18^F-FDG PET/CT Studies

The ^18^F-FDG PET/CT scans at diagnosis, and at recovery when available, were acquired at Sant’Andrea University Hospital of Rome (Rome, Italy), International European Oncology hospital in Milan (Milan, Italy), and University Medical Centre Groningen (Groningen, The Netherlands) by using a hybrid PET/CT system (Siemens, Germany).

The ^18^F-FDG (3–5 MBq/Kg) was administered 1 h before imaging from pelvis to head as per standardized EARL procedures [50,51]. After image acquisition, attenuation corrected PET images were automatically fused with CT images and displayed in maximum intensity projection (MIP) in axial, coronal and sagittal plane.

To minimize mismatches between CT and PET scans, CT images were obtained with a slice thickness of 3.75 mm. Moreover, immediately after CT acquisition, PET scan started from the pelvic region (therefore, less than 2 min delay from CT to pelvic PET).

Patient data were retrospectively collected via electronic patient files. Given the retrospective nature of this study, the local Ethical Committee waived the need for approval, but written informed consent was obtained from all non-ICU patients. For ICU patients, written informed consent was obtained from their closest relatives.

### 2.3. Analysis of ^18^F-FDG PET/CT Studies

A visual analysis of ^18^F-FDG metabolic activity on lungs and mediastinal lymph-nodes was performed to assess the severity of the infection [43,52,53,54,55] and to meet the clinical need of each patient. The ^18^F-FDG biodistribution in both thyroid and adrenal glands was retrospectively visually evaluated.

A semi-quantitative analysis on axial sections of EARL reconstructed images was also performed by drawing circular regions of interest (ROIs) in right and left thyroid lobe and on both adrenal glands and by calculating the maximum and mean standardized uptake value (SUVmax and SUVmean). The whole glands’ activity was calculated as the mean of right and left lobe of thyroid gland and as the mean of left and right adrenal gland. In patients with the right adrenal gland too close to the liver, the measurement was not performed due to overlap of liver metabolic activity.

### 2.4. Statistical Analysis

Continuous variables are presented as mean ± standard deviation (SD) and 95% confidence interval (95% CI).

Comparisons between control subjects, COVID-19 at diagnosis and COVID-19 ICU of SUVmax and SUVmean of thyroid and adrenal glands were evaluated by generalized linear mixed model (GLIMMIX) with Gaussian distribution.

Normality residuals were tested by Shapiro–Wilk test and checking the Q-Q plot. Homoscedasticity was evaluated by checking the studentized residuals vs. fitted values plot.

Post-hoc analysis was performed using the Tukey method.

The differences between thyroid and adrenal glands in control subjects versus COVID-19 subjects after recovery were estimated by the Student’s *t*-test (normality verified) or Brunner–Munzel test (normality failed).

COVID-19 at diagnosis versus COVID-19 after recovery of thyroid and adrenal glands was tested by paired Student’s *t*-test.

The cut-offs of thyroid and adrenal gland uptake were obtained using Youden index, as described elsewhere [56,57].

Statistical analysis was performed using the SAS v.9.4 TS level 1M8 (SAS Institute Inc., Cary, NC, USA). A *p*-value < 0.05 was considered statistically detectable.

## 3. Results

### 3.1. Patients

Between March 2020 and March 2022, we enrolled 33 patients (twenty-four males and nine females, mean age 57.67 ± 14 years) with SARS-CoV-2 infection admitted to three centres and studied with ^18^F-FDG PET/CT (fourteen patients were studied at Sant’Andrea hospital in Rome; four patients were recruited by IEO in Milan; and fifteen patients were studied at UMC Groningen). Five out of these fifteen patients were mechanically ventilated and admitted to ICU for their critical illness at the time of ^18^F-FDG PET/CT.

Seven patients were studied twice, at diagnosis and after complete clinical recovery, which occurred between 3 and 6 months after diagnosis, and a double negative RT-PCR test for SARS-CoV-2.

Respiratory symptoms (mainly dyspnoea), anosmia and persistent fever were the main causes for hospitalization of these patients. These clinical manifestations appeared on average 5 to 7 days before ^18^F-FDG PET/CT. Therapies varied amongst the different centres and according to the severity of patients’ clinical manifestations, and mainly included personalized doses of corticosteroids, remdesivir, tocilizumab, paracetamol and hydroxychloroquine.

One critically ill patient admitted in ICU died due to progressive respiratory failure and extra-pulmonary organ failure. Another five patients died for unrelated complications.

For the control group, we identified 36 age-matched patients (17 males and 19 females, mean age 59.40 ± 15.73 years) without known thyroid and adrenal dysfunctions that underwent ^18^F-FDG PET/CT for oncological and non-oncological reasons and who showed no pathologic metabolic lesions at scan analysis.

### 3.2. Thyroid and Adrenal Gland Analysis

Semi-quantitative results of thyroid and adrenal gland ^18^F-FDG uptake in control group (group A), non-critical COVID-19 patients (group B) and ICU group (group C) are reported in Table 1.

**Figure 1:**
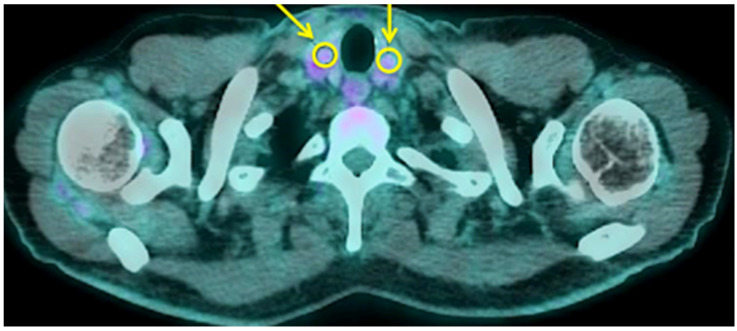
Example of calculation of quantitative parameters on thyroid gland of one COVID-19 patient. Yellow circles and arrows show the regions of interest drawn on thyroid tissue. In this patient, the right lobe had an SUVmax of 2.18 and an SUVmean of 2.05; the left lobe had an SUVmax of 2.10 and an SUVmean of 1.94.

When examining thyroid ^18^F-FDG uptake (Figure 1), no differences were observed among the three groups of patients in terms of pattern distribution. Non-critical patients showed slightly higher SUVmax and SUVmean compared to normal subjects.

No statistically significant differences were observed comparing basal and follow-up study in the seven patients who repeated PET/CT scan, but interestingly, they showed higher SUVmax (*p* = 0.009) and SUVmean (*p* = 0.004) at follow-up study compared to control subjects (Table 2). Despite mean values that were significantly higher at follow-up scan, only three patients showed an increased thyroid uptake, thus suggesting that thyroid inflammation or thyroid hyperfunction may appear evident several months after COVID-19 diagnosis, but not in all patients.

**Figure 2:**
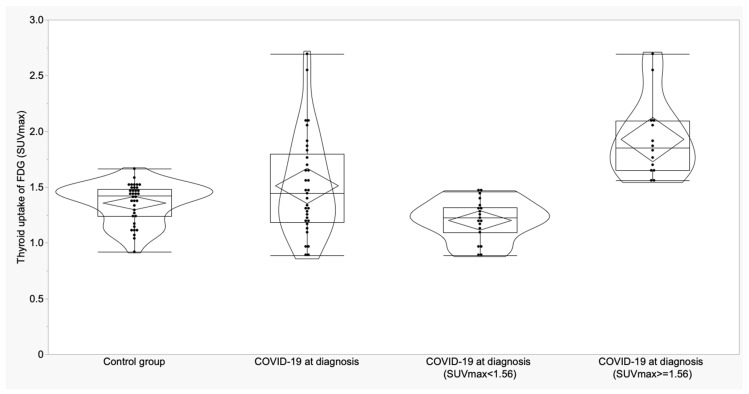
Boxplots and violin plots of SUVmax in thyroid glands of control group vs. COVID-19 patients at the diagnosis and distribution of SUVmax of COVID-19 patients according to the cut-off.

**Figure 3:**
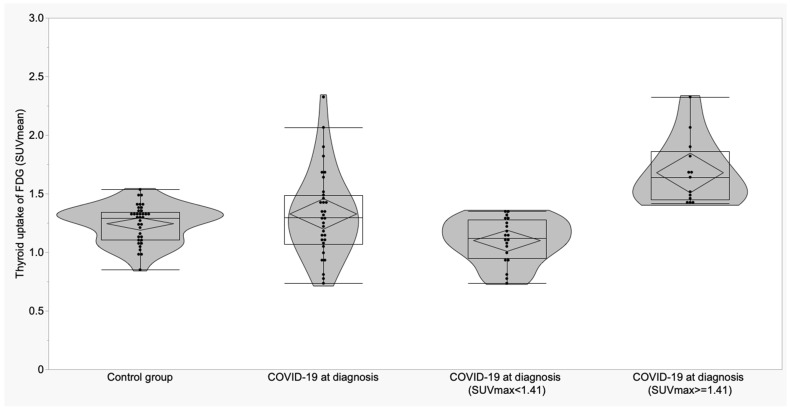
Boxplots and violin plots of SUVmean in thyroid glands of control group vs. COVID-19 patients at the diagnosis and distribution of SUVmean of COVID-19 patients according to the cut-off.

For thyroid gland in control subjects, a cut-off of 1.56 for SUVmax showed a sensitivity of 42.4% and a specificity of 94.4%; for SUVmean, a cut-off of 1.41 provided 39.4% of sensitivity and 91.7% of specificity. SUVmax was higher than the cut-off in 14/33 COVID-19 patients at diagnosis (42.4%) and in 2/36 controls (5.56%) (*p* = 0.0003). SUVmean was higher than the cut-off in 13/33 COVID-19 patients at diagnosis (39.4%) and in 3/36 controls (8.3%) (*p* = 0.002) (Figure 2 and Figure 3).

Only two patients were excluded from the analysis of right gland because it was too close to the liver.

**Figure 4:**
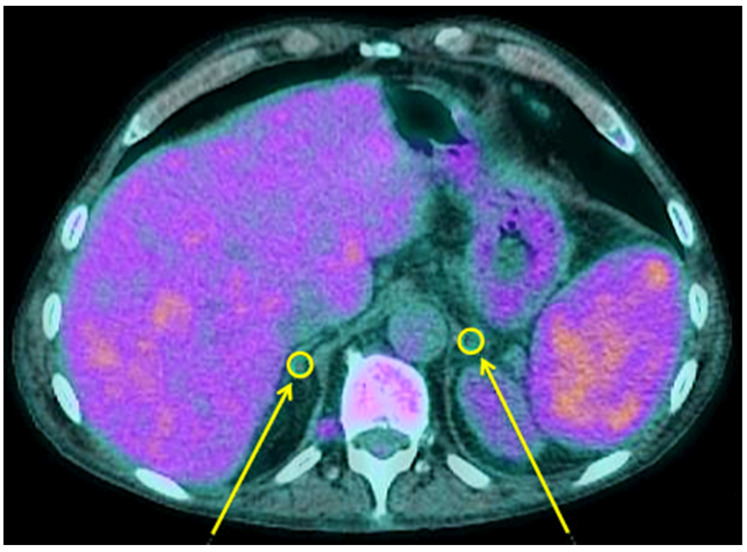
Example of calculation of quantitative parameters on adrenal glands of one COVID-19 patient. Yellow circles and arrows show the regions of interest. In this patient, the right adrenal gland had an SUVmax of 1.33 and an SUVmean of 1.21; the left adrenal gland had an SUVmax of 1.26 and an SUVmean of 1.20.

Similar to the thyroid gland, no correlation was detected between the uptake distribution pattern of adrenal glands and disease severity (Figure 4). As reported in Table 1, the adrenal glands of COVID-19 patients, either admitted in low-intensity unit or in ICU, showed statistically lower SUVmax and SUVmean than control subjects.

The post-hoc analysis showed lower SUVmax and SUVmean in patients with moderate infection (group B; *p* < 0.0001 for both SUVmax and SUVmean) and in ICU patients (group C; *p* = 0.003 for SUVmax and *p* = 0.03 for SUVmean) compared to the control group.

No statistically significant differences were observed when comparing the basal study and the follow-up study in the seven patients who repeated PET/CT scan, but after recovery, they showed persistently lower ^18^F-FDG uptake in adrenal glands compared to healthy control subjects, in terms of SUVmax and SUVmean (Table 3).

For adrenal glands, a cut-off of 1.99 for SUVmax showed a sensitivity of 93.9% and specificity of 66.7%; for SUVmean, a cut-off of 1.88 provided 97% of sensitivity and 52.8% of specificity (Figure 3 and Figure 4). SUVmax was lower than the cut-off in 31/33 COVID-19 patients at diagnosis (93.9%) and in 12/36 controls (33.3%) (*p* < 0.0001).

**Figure 5:**
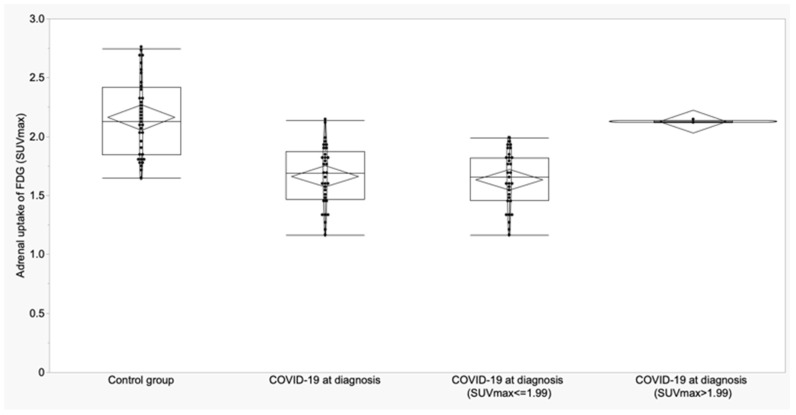
Boxplots and violin plots of SUVmax in adrenal glands of control group vs. COVID-19 patients at the diagnosis and distribution of SUVmax of COVID-19 patients according to the cut-off.

**Figure 6:**
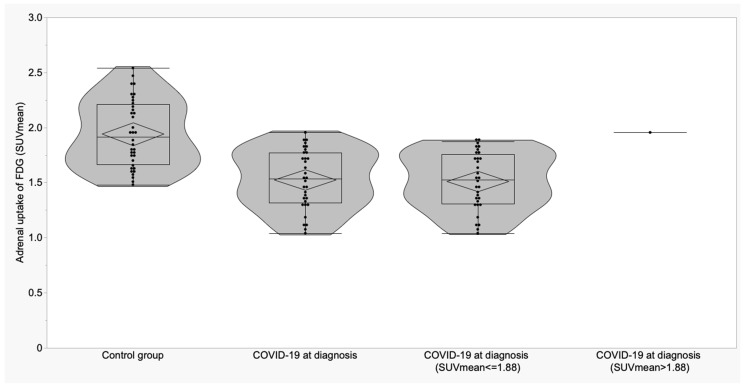
Boxplots and violin plots of SUVmean in adrenal glands of control group vs. COVID-19 patients at the diagnosis and distribution of SUVmean of COVID-19 patients according to the cut-off.

SUVmean was lower than the cut-off in all but one of the COVID-19 patients at diagnosis (97%) and in 17/36 controls (47.2%) (*p* < 0.0001) (Figure 5 and Figure 6).

## 4. Discussion

Endocrine glands represent a potential target of SARS-CoV-2 infection, and thyroid dysfunctions and adrenal insufficiency have been largely described in the literature [4,5,6,7,8,9,10,11,12,13,14,15,16,17,18,19,20,21,22,23,24,25,26,27,58,59,60,61]. Their impairment takes part of the complex and variegated symptomatology experienced by the patients and might influence the clinical course of the disease [62].

It is well known that this virus is able to trigger long-term immune activation thus being responsible for latent or overt inflammatory and autoimmune phenomena or hypofunctionality [63,64]. Drugs may also indirectly impair endocrine glands by exerting a suppressive effect, and this especially, holds true for HPA axis due to the large use of steroids, which may turn in the development of hypocortisolism [23,27,28,29]. It is, indeed well known that a prolonged treatment with high corticosteroid doses leads to high risk of adrenal insufficiency that may take up to 6 months to revert [29]. In COVID-19 patients, both the virus itself and steroid treatment could contribute to the development of cortisol deficiency and to understand which of these aspects has a major role, deserves further speculations.

As opposed to thyroid dysfunctions, which usually revert after a variable time from the infection, adrenal insufficiency might persist for several months after the recovery, thus having a potential role in long-COVIDsyndrome. In particular, HPA axis impairment and hypothyroidism are, in part, implied in persistent fatigue reported by the majority of patient with long-COVID [58,59,60,61,62].

Although ^18^F-FDG PET/CT plays only a marginal role in the diagnosis and monitoring of COVID-19 patients, the increasing evidence of incidental findings detected by this imaging modality further supports the awareness that SARS-CoV-2 infection is not only confined to the respiratory tract, but rather it may involve several other organs and apparatuses [36,37,38,39,40,41,42,43,65,66].

Therefore, this retrospective multicentre study aimed at comparing the ^18^F-FDG uptake in thyroid and adrenal glands in patients affected by SARS-CoV-2 infection and in normal control subjects. Moreover, we compared the ^18^F-FDG uptake in seven patients who performed an ^18^F-FDG PET/CT study at both basal time and after recovery.

For thyroid gland we did not observe any difference in ^18^F-FDG uptake between control subjects and newly diagnosed COVID-19 patients (either admitted in low-intensity departments or in ICU). Neither was a difference observed from basal PET/CT scan and follow-up study in the subgroup of patients who performed a double scan. However, we found statistically higher metabolic activity in the follow-up PET/CT as compared to control group, thus potentially suggesting an inflammatory status or hyperfunction of thyroid gland. Unfortunately, given the retrospective nature of this study, data on thyroid function were not collected; therefore, we are not able to correlate ^18^F-FDG uptake with the hormonal status of these patients. It would have been very interesting to investigate whether these patients experienced a thyroiditis or changes in thyroid hormone function during and after SARS-CoV-2 infection.

As far as adrenal glands are concerned, we found interesting results. COVID-19 patients (either admitted in low-intensity departments or in ICU) showed statistically lower SUVmax and SUVmean than control subjects. No differences were observed from basal PET/CT scan and follow-up study, the metabolic activity did not normalize at recovery but was persistently lower than activity observed in healthy controls. This would potentially suggest a suppressive state of adrenal glands, which may persist for a long time after recovery, thus negatively influencing patients’ quality of life [67]. As previously mentioned, given the observational retrospective nature of this study, we are not able to correlate PET/CT findings with pituitary and adrenal function, but this aspect deserves further speculation, especially considering the role of adrenal insufficiency in the development of long-COVID syndrome. Furthermore, we are not able to determine the contribution of direct gland damage due to the virus or the possible role of steroid therapies in adrenal glands of COVID-19 patients. We can only speculate that in our population, a combination of both aspects would have contributed to low ^18^F-FDG uptake in adrenal glands, but this requires further investigations.

Similar to our findings, Sollini et al. recently analysed ^18^F-FDG uptake in several organs in patients suffering from long-COVID, reporting lower adrenal uptake in COVID-19 patients than in control subjects and speculating that hypocortisolism might play a role in long-COVID syndrome [68].

Conversely, Bülbül and colleagues recently assessed ^18^F-FDG uptake in several endocrine glands including pituitary, thyroid, adrenal glands, testis and pancreas. Although they found a statistically higher SUVmean in the pancreas of COVID-19 patients compared to non-COVID-19 patients, they did not observe statistical differences in thyroid, adrenal, pituitary glands and testis [69].

Despite the multicentre nature, our study has several limitations. First of all, as previously mentioned, it has a retrospective design. All ^18^F-FDG PET/CT scans were performed during the pandemic wave and, in that period, the restrictions due to spreading of SARS-CoV-2 infection in the many departments had a negative impact on the working quality also in Nuclear Medicine Units and did not allow the clinician to deeply assess the whole status of the patients [70,71,72,73].

Moreover, the number of examined patients is limited; however, ^18^F-FDG PET/CT is not included as a crucial imaging modality for the assessment of COVID-19 severity, rather it retains a supportive role over conventional radiological scans, and it is justified only in selected patients. Indeed, despite the large number of COVID-19 patients admitted in our three centres during the pandemic waves, only a small percentage of them required a PET/CT study to assess the severity of the infection, to detect possible involvement of other organs or when clinical symptoms did not match CT findings. Another limitation is the lack of pituitary, thyroid and adrenal function assessment in COVID-19 patients. We, therefore, herein only describe the ^18^F-FDG uptake in thyroid and adrenal gland, opening new questions about the different behaviour of different endocrine glands. Some may show an inflammatory or autoimmune status and hyper-functionality; some others may be downregulated and lead to hypofunctionality. The availability of laboratory tests would have allowed a correlation between hormonal status of these patients and ^18^F-FDG uptake and would have laid the basis for further speculations. Moreover, we cannot exclude that the use of high-dose glucocorticoids, which has been largely adopted during the SARS outbreak, might be responsible of the reduced metabolism observed in adrenal glands.

Despite these limitations, our observation should alert clinicians about excluding a cortisol deficiency or a thyroid impairment in these kinds of patients in order to promptly start supportive therapies [23,74].

The correlation between hormonal status of COVID-19 patients, in particular on the HPA axis and ^18^F-FDG uptake deserves further speculations and might be relevant in preventing long-COVID syndrome and improving patients’ quality of life.

## 5. Conclusions

Despite the observation that thyroid uptake was comparable to normal subjects at disease onset, after recovery, a subgroup of patients showed an increased metabolic activity, thus possibly suggesting the onset of an inflammatory thyroiditis. Moreover, we observed lower ^18^F-FDG uptake in adrenal glands of COVID-19 patients compared to normal subjects and this finding did not normalize after recovery. This would potentially suggest a persistent hypofunctionality and would alert clinicians to investigate the endocrine status at both basal time and after the recovery. In the long-COVID era, this aspect should be one of the priority areas for future research.

## Figures and Tables

**Table 1:** Differences between control group, non-critical COVID-19 patients and the ICU group, in terms of SUVmax and SUVmean of thyroid and adrenal glands.

Variable	A Control Subjects (n = 36) Mean ± SD (95% CI)	B COVID-19 at Diagnosis (n = 28) Mean ± SD (95% CI)	C COVID-19 ICU (n = 5) Mean ± SD (95% CI)	*p*	*p* (A vs. B)	*p* (A vs. C)	*p* (B vs. C)
Thyroid (SUVmax)	1.36 ± 0.18 (1.30 to 1.42)	1.55 ± 0.47 (1.37 to 1.73)	1.31 ± 0.24 (1.02 to 1.61)	0.06	--	--	--
Thyroid (SUVmean)	1.24 ± 0.16 (1.19 to 1.30)	1.35 ± 0.38 (1.21 to 1.50)	1.18 ± 0.22 (0.90 to 1.45)	0.19	--	--	--
Adrenal (SUVmax)	2.16 ± 0.32 (2.05 to 2.27)	1.65 ± 0.26 (1.55 to 1.75)	1.73 ± 0.26 (1.40 to 2.06)	<0.0001	<0.0001	0.003	0.58
Adrenal (SUVmean)	1.94 ± 0.30 (1.84 to 2.04)	1.51 ± 0.27 (1.40 to 1.61)	1.64 ± 0.23 (1.35 to 1.93)	<0.0001	<0.0001	0.03	0.34

**Table 2:** Differences between control group and COVID-19 patients after recovery in terms of SUVmax and SUVmean of thyroid gland.

Thyroid Uptake	Control Group (n = 36) Mean ± SD (95% CI)	COVID-19 after Recovery (n = 7) Mean ± SD (95% CI)	*p*
SUVmax	1.36 ± 0.18 (1.30 to 1.42)	1.65 ± 0.30 (1.37 to 1.93)	0.009
SUVmean	1.24 ± 0.16 (1.19 to 1.30)	1.46 ± 0.22 (1.25 to 1.66)	0.004

**Table 3:** Differences between control group and COVID-19 patients after recovery in terms of SUVmax, SUVmean of adrenal glands.

Adrenal Glands’ Uptake	Control Group (n = 36) Mean ± SD (95% CI)	COVID-19 after Recovery (n = 7) Mean ± SD (95% CI)	*p*
SUVmax	2.16 ± 0.32 (2.05 to 2.27)	1.66 ± 0.26 (1.57 to 1.75)	<0.0001
SUVmean	1.94 ± 0.30 (1.84 to 2.04)	1.53 ± 0.26 (1.43 to 1.62)	<0.0001

## Data Availability

Data are available upon request.

## References

[1] Huang C., Wang Y., Li X., Ren L., Zhao J., Hu Y., Zhang L., Fan G., Xu J., Gu X. (2020). Clinical features of patients infected with 2019 novel coronavirus in Wuhan, China. Lancet.

[2] Chen N., Zhou M., Dong X., Qu J., Gong F., Han Y., Qiu Y., Wang J., Liu Y., Wei Y. (2020). Epidemiological and clinical characteristics of 99 cases of 2019 novel coronavirus pneumonia in Wuhan, China: A descriptive study. Lancet.

[3] Hossain M.F., Hasana S., Mamun A.A., Uddin M.S., Wahed M.I.I., Sarker S., Behl T., Ullah I., Begum Y., Bulbul I.J. (2020). COVID-19 outbreak: Pathogenesis, current therapies, and potentials for future management. Front. Pharmacol..

[4] Giovanella L., Ruggeri R.M., Ovčariček P.P., Campenni A., Treglia G., Deandreis D. (2021). Prevalence of thyroid dysfunction in patients with COVID-19: A systematic review. Clin. Transl. Imaging.

[5] Marazuela M., Giustina A., Puig-Domingo M. (2020). Endocrine and metabolic aspects of the COVID-19 pandemic. Rev. Endocr. Metab. Disord..

[6] Parolin M., Parisotto M., Zanchetta F., Sartorato P., De Menis E. (2021). Coronaviruses and endocrine system: A systematic review on evidence and shadows. Endocr. Metab. Immune Disord. Drug Targets.

[7] Clarke S.A., Abbara A., Dhillo W.S. (2021). Impact of COVID-19 on the Endocrine System: A Mini-review. Endocrinology.

[8] Chen M., Zhou W., Xu W. (2021). Thyroid function analysis in 50 patients with COVID-19: A retrospective study. Thyroid.

[9] Khoo B., Tan T., Clarke S.A., Mills E.G., Patel B., Modi M., Phylactou M., Eng P.C., Thurston L., Alexander E.C. (2021). Thyroid function before, during, and after COVID-19. J. Clin. Endocrinol. Metab..

[10] Lui D.T.W., Lee C.H., Chow W.S., Lee A.C.H., Tam A.R., Fong C.H.Y., Law C.Y., Leung E.K.H., To K.K.W., Tan K.C.B. (2021). Thyroid dysfunction in relation to immune profile, disease status, and outcome in 191 patients with COVID-19. J. Clin. Endocrinol. Metab..

[11] Muller I., Cannavaro D., Dazzi D., Covelli D., Mantovani G., Muscatello A., Ferrante E., Orsi E., Resi V., Longari V. (2020). SARS-CoV-2-related atypical thyroiditis. Lancet Diabetes Endocrinol..

[12] Lania A., Sandri M.T., Cellini M., Mirani M., Lavezzi E., Mazziotti G. (2020). Thyrotoxicosis in patients with COVID-19: The THYRCOV study. Eur. J. Endocrinol..

[13] Mateu-Salat M., Urgell E., Chico A. (2020). SARS-COV-2 as a trigger for autoimmune disease: Report of two cases of Graves’ disease after COVID-19. J. Endocrinol. Investig..

[14] Jiménez-Blanco S., Pla-Peris B., Marazuela M. (2021). COVID-19: A cause of recurrent Graves’ hyperthyroidism?. J. Endocrinol. Investig..

[15] Morita S., Takagi T., Inaba H., Furukawa Y., Kishimoto S., Uraki S., Shimo N., Takeshima K., Uraki S., Doi K. (2023). Effect of SARS-CoV-2 BNT162b2 mRNA vaccine on thyroid autoimmunity: A twelve-month follow-up study. Front. Endocrinol..

[16] Muller I., Consonni D., Crivicich E., Di Marco F., Currò N., Salvi M. (2023). Increased risk of Thyroid Eye Disease following Covid-19 Vaccination. J. Clin. Endocrinol. Metab..

[17] Mainieri F., Chiarelli F., Betterle C., Bernasconi S. (2023). Graves’ disease after COVID mRNA vaccination for the first time diagnosed in adolescence-case report. Cause and effect relationship or simple coincidence?. J. Pediatr. Endocrinol. Metab..

[18] Kumar R., Guruparan T., Siddiqi S., Sheth R., Jacyna M., Naghibi M., Vrentzou E. (2020). A case of adrenal infarction in a patient with COVID 19 infection. BJR Case Rep..

[19] Elkhouly M.M.N., Elazzab A.A., Moghul S.S. (2021). Bilateral adrenal hemorrhage in a man with severe COVID-19 pneumonia. Radiol. Case Rep..

[20] Sharrack N., Baxter C.T., Paddock M., Uchegbu E. (2020). Adrenal haemorrhage as a complication of COVID-19 infection. BMJ Case Rep..

[21] Álvarez-Troncoso J., Zapatero Larrauri M., Montero Vega M.D., Vallano R.G., Pelaez E.P., Rojas-Marcos P.M., Martin-Luengo F., Del Campo P.L., Gil C.R.H., Esteban E.T. (2020). Case report: COVID-19 with bilateral adrenal hemorrhage. Am. J. Trop. Med. Hyg..

[22] Hashim M., Athar S., Gaba W.H. (2021). New onset adrenal insufficiency in a patient with COVID-19. BMJ Case Rep..

[23] Pal R., Banerjee M. (2020). COVID-19 and the endocrine system: Exploring the unexplored. J. Endocrinol. Investig..

[24] Wheatland R. (2004). Molecular mimicry of ACTH in SARS-implications for corticosteroid treatment and prophylaxis. Med. Hypotheses.

[25] Mao Y., Xu B., Guan W., Xu D., Li F., Ren R., Zhu X., Gao Y., Jiang L. (2021). The Adrenal Cortex, an Underestimated Site of SARS-CoV-2 Infection. Front. Endocrinol..

[26] Piticchio T., Le Moli R., Tumino D., Frasca F. (2021). Relationship between betacoronaviruses and the endocrine system: A new key to understand the COVID-19 pandemic—A comprehensive review. J. Endocrinol. Investig..

[27] Lisco G., De Tullio A., Stragapede A., Solimando A.G., Albanese F., Capobianco M., Giagulli V.A., Guastamacchia E., de Pergola G., Vacca A. (2021). COVID-19 and the Endocrine System: A Comprehensive Review on the Theme. J. Clin. Med..

[28] Brender E., Lynm C., Glass R.M. (2005). JAMA patient page. Adrenal insufficiency. JAMA.

[29] Broersen L.H., Pereira A.M., Jørgensen J.O., Dekkers O.M. (2015). Adrenal Insufficiency in Corticosteroids Use: Systematic Review and Meta-Analysis. J. Clin. Endocrinol. Metab..

[30] Davis H.E., Assaf G.S., McCorkell L., Wei H., Low R.J., Re’em Y., Redfield S., Austin J.P., Akrami A. (2021). Characterizing long COVID in an international cohort: 7 months of symptoms and their impact. EClinicalMedicine.

[31] Ladds E., Rushforth A., Wieringa S., Taylor S., Rayner C., Husain L., Greenhalgh T. (2020). Persistent symptoms after Covid-19: Qualitative study of 114 “long Covid” patients and draft quality principles for services. BMC Health Serv. Res..

[32] Mendelson M., Nel J., Blumberg L., Madhi S.A., Dryden M., Stevens W. (2020). Long-COVID: An evolving problem with an extensive impact. S. Afr. Med. J..

[33] Lopez-Leon S., Wegman-Ostrosky T., Perelma C., Sepulveda R., Rebolledo P.A., Cuapio A., Villapol S. (2021). More than 50 long-term effects of COVID-19: A systematic review and meta-analysis. Sci. Rep..

[34] Clarke S.A., Phylactou M., Patel B., Mills E.G., Muzi B., Izzi-Engbeaya C., Choudhury S., Khoo B., Meeran K., Comninos A.N. (2021). Normal adrenal and thyroid function in patients who survive COVID-19 infection. J. Clin. Endocrinol. Metab..

[35] Salzano C., Saracino G., Cardillo G. (2021). Possible adrenal involvement in long COVID syndrome. Medicina.

[36] Olivari L., Riccardi N., Rodari P., Buonfrate D., Diodato S., Formenti F., Angheben A., Salgarello M. (2020). Accidental diagnosis of COVID-19 pneumonia after 18F FDG PET/CT: A case series. Clin. Transl. Imaging.

[37] Albano D., Bertagna F., Bertoli M., Bosio G., Lucchini S., Motta F., Panarotto M.B., Peli A., Camoni L., Bengel F.M. (2020). Incidental findings suggestive of COVID-19 in asymptomatic patients undergoing nuclear medicine procedures in a high-prevalence region. J. Nucl. Med..

[38] Setti L., Kirienko M., Dalto S.C., Bonacina M., Bombardieri E. (2020). FDG-PET/CT findings highly suspicious for COVID-19 in an Italian case series of asymptomatic patients. Eur. J. Nucl. Med. Mol. Imaging.

[39] Qin C., Liu F., Yen T.C., Lan X. (2020). 18F-FDG PET/CT findings of COVID-19: A series of four highly suspected cases. Eur. J. Nucl. Med. Mol. Imaging.

[40] Colandrea M., Gilardi L., Travaini L.L., Fracassi S.L.V., Funicelli L., Grana C.M. (2020). 18F-FDG PET/CT in asymptomatic patients with COVID-19: The submerged iceberg surfaces. Jpn. J. Radiol..

[41] Bai Y., Xu J., Chen L., Fu C., Kang Y., Zhang W., Fakhri G.E., Gu J., Shao F., Wang M. (2021). Inflammatory response in lungs and extrapulmonary sites detected by [18F] fluorodeoxyglucose PET/CT in convalescing COVID-19 patients tested negative for coronavirus. Eur. J. Nucl. Med. Mol. Imaging.

[42] Signore A., Lauri C., Bianchi M.P., Pelliccia S., Lenza A., Tetti S., Martini M.L., Franchi G., Trapasso F., De Biase L. (2022). [18F]FDG PET/CT in Patients Affected by SARS-CoV-2 and Lymphoproliferative Disorders and Treated with Tocilizumab. J. Pers. Med..

[43] Signore A., Lauri C., Colandrea M., Di Girolamo M., Chiodo E., Grana C.M., Campagna G., Aceti A. (2022). Lymphopenia in patients affected by SARS-CoV-2 infection is caused by margination of lymphocytes in large bowel: An [18F]FDG PET/CT study. Eur. J. Nucl. Med. Mol. Imaging.

[44] Nawwar A.A., Searle J., Hagan I., Lyburn I.D. (2021). COVID-19 vaccination induced axillary nodal uptake on [18F] FDG PET/CT. Eur. J. Nucl. Med. Mol. Imaging.

[45] Nawwar A.A., Searle J., Hopkins R., Lyburn I.D. (2021). False-positive axillary lymph nodes on FDG PET/CT resulting from COVID-19 immunization. Clin. Nucl. Med..

[46] Doss M., Nakhoda S.K., Li Y., Jian Q.Y. (2021). COVID-19 vaccine–related local FDG uptake. Clin. Nucl. Med..

[47] Eifer M., Eshet Y. (2021). Imaging of COVID-19 vaccination at FDG PET/CT. Radiology.

[48] McIntosh L.J., Bankier A.A., Vijayaraghavan G.R., Licho R., Rosen M.P. (2021). COVID-19 vaccination-related uptake on FDG PET/CT: An emerging dilemma and suggestions for management. Am. J. Roentgenol..

[49] Moghimi S., Wilson D., Martineau P. (2021). FDG PET Findings Post–COVID Vaccinations: Signs of the Times?. Clin. Nucl. Med..

[50] Jamar F., Buscombe J., Chiti A., Christian P.E., Delbeke D., Donohoe K.J., Signore A. (2013). EANM/SNMMI guideline for 18F-FDG use in inflammation and infection. J. Nucl. Med..

[51] Boellaard R., Delgado-Bolton R., Oyen W.J., Giammarile F., Tatsch K., Eschner W., Verzijlbergen F.J., Barrington S.F., Pike L.C., Weber W.A. (2015). FDG PET/CT: EANM procedure guidelines for tumour imaging: Version 2.0. Eur. J. Nucl. Med. Mol. Imaging.

[52] Inoue K., Goto R., Okada K., Kinomura S., Fukuda H. (2009). A bone marrow F-18 FDG uptake exceeding the liver uptake may indicate bone marrow hyperactivity. Ann. Nucl. Med..

[53] Ahn S.S., Hwang S.H., Jung S.M., Lee S.-W., Park Y.-B., Yun M., Song J.J. (2017). Evaluation of spleen glucose metabolism using 18F-FDG PET/CT in patients with febrile autoimmune disease. J. Nucl. Med..

[54] Ahn S.S., Hwang S.H., Jung S.M., Lee S.-W., Park Y.-B., Yun M., Song J.J. (2017). The clinical utility of splenic fluorodeoxyglucose uptake for diagnosis and prognosis in patients with macrophage activation syndrome. Medicine.

[55] Boursier C., Duval X., Mahida B., Hoen B., Goehringer F., Selton-Suty C., Chevalier E., Roch V., Lamiral Z., Bourdon A. (2021). Hypermetabolism of the spleen or bone marrow is an additional albeit indirect sign of infective endocarditis at FDG-PET. J. Nucl. Cardiol..

[56] Youden W.J. (1950). Index for rating diagnostic tests. Cancer.

[57] Perkins N.J., Schisterman E.F. (2005). The Youden Index and the optimal cut-point corrected for measurement error. Biom. J..

[58] Alzahrani A.S., Mukhtar N., Aljomaiah A., Aljamei H., Bakhsh A., Alsudani N., Elsayed T., Alrashidi N., Fadel R., Alqahtani E. (2021). The Impact of COVID-19 Viral Infection on the Hypothalamic-Pituitary-Adrenal Axis. Endocr. Pract..

[59] Siejka A., Barabutis N. (2021). Adrenal insufficiency in the COVID-19 era. Am. J. Physiol. Endocrinol. Metab..

[60] Akbas E.M., Akbas N. (2021). COVID-19, adrenal gland, glucocorticoids, and adrenal insufficiency. Biomed. Pap. Med. Fac. Univ. Palacky. Olomouc Czech Repub..

[61] Kanczkowski W., Evert K., Stadtmüller M., Haberecker M., Laks L., Chen L.S., Frontzek K., Pablik J., Hantel C., Beuschlein F. (2022). COVID-19 targets human adrenal glands. Lancet Diabetes Endocrinol..

[62] Daraei M., Hasibi M., Abdollahi H., Mirabdolhagh Hazaveh M., Zebaradst J., Hajinoori M., Asadollahi-Amin A. (2020). Possible role of hypothyroidism in the prognosis of COVID-19. Intern. Med. J..

[63] Acosta-Ampudia Y., Monsalve D.M., Rojas M., Rodríguez Y., Zapata E., Ramírez-Santana C., Anaya J.M. (2022). Persistent autoimmune activation and proinflammatory state in post-coronavirus disease 2019 syndrome. J. Infect. Dis..

[64] Mongioì L.M., Barbagallo F., Condorelli R.A., Cannarella R., Aversa A., La Vignera S., Calogero A.E. (2020). Possible long-term endocrine-metabolic complications in COVID-19: Lesson from the SARS model. Endocrine.

[65] Albano D., Treglia G., Giovanella L., Giubbini R., Bertagna F. (2020). Detection of thyroiditis on PET/CT imaging: A systematic review. Hormones.

[66] Van Leer B., van Snick J.H., Londema M., Nijsten M.W.N., Kasalak O., Slart R.H.J.A., Glaudemans A.W.J.M., Pillay J. (2023). [18F]FDG-PET/CT in mechanically ventilated critically ill patients with COVID-19 ARDS and persistent inflammation. Clin. Transl. Imaging.

[67] Frara S., Allora A., Castellino L., di Filippo L., Loli P., Giustina A. (2021). COVID-19 and the pituitary. Pituitary.

[68] Sollini M., Morbelli S., Ciccarelli M., Cecconi M., Aghemo A., Morelli P., Chiola S., Gelardi F., Chiti A. (2021). Long COVID hallmarks on [18F] FDG-PET/CT: A case-control study. Eur. J. Nucl. Med. Mol. Imaging.

[69] Bülbül O., Göksel S., Demet N.A.K. (2023). Effect of Coronavirus Disease 2019 on Fluorine-18 fluorodeoxyglucose Uptake of Endocrine Organs. Cumhur. Med. J..

[70] Annunziata S., Bauckneht M., Albano D., Argiroffi G., Calabro D., Abenavoli E., Linguanti F., Laudicella R., Young Committee of the Italian Association of Nuclear Medicine (AIMN) (2020). Impact of the COVID-19 pandemic in nuclear medicine departments: Preliminary report of the first international survey. Eur. J. Nucl. Med. Mol. Imaging.

[71] Freudenberg L.S., Paez D., Giammarile F., Cerci J., Modiselle M., Pascual T.N.B., El-Haj N., Orellana P., Pynda Y., Carrio I. (2020). Global impact of COVID-19 on nuclear medicine departments: An international survey in April 2020. J. Nucl. Med..

[72] Freudenberg L.S., Dittmer U., Herrmann K. (2020). Impact of COVID-19 on nuclear medicine in Germany, Austria and Switzerland: An international survey in April 2020. Nuklearmedizin.

[73] Annunziata S., Albano D., Laudicella R., Bauckneht M., Young Committee of the Italian Association of Nuclear Medicine (AIMN) (2020). Surveys on COVID-19 in nuclear medicine: What happened and what we learned. Clin. Transl. Imaging.

[74] Pal R. (2020). COVID-19, hypothalamo-pituitary-adrenal axis and clinical implications. Endocrine.

